# Are formalised implementation activities associated with aspects of quality of care in general practice? A cross-sectional study

**DOI:** 10.3399/bjgpopen17X100737

**Published:** 2017-04-05

**Authors:** Jette V Le, Jesper Lykkegaard, Line B Pedersen, Helle Riisgaard, Jørgen Nexøe, Jeanette Lemmergaard, Jens Søndergaard

**Affiliations:** 1 Postdoctoral Fellow, Research Unit of General Practice, Department of Public Health, University of Southern Denmark, Odense, Denmark; 2 Postdoctoral Fellow and GP, Research Unit of General Practice, Department of Public Health, University of Southern Denmark, Odense, Denmark; 3 Postdoctoral Fellow, Research Unit of General Practice, Department of Public Health and COHERE, Department of Business Economics, University of Southern Denmark, Odense, Denmark; 4 Research Unit of General Practice, Department of Public Health, University of Southern Denmark, Odense, Denmark; 5 Associate Professor and GP, Research Unit of General Practice, Department of Public Health, University of Southern Denmark, Odense, Denmark; 6 Associate Professor, Department of Marketing & Management, University of Southern Denmark, Odense, Denmark; 7 Professor and GP, Research Unit of General Practice, Department of Public Health, University of Southern Denmark, Odense, Denmark

**Keywords:** general practice, quality of health care, evidence-based practice, organization and administration, cross-sectional study

## Abstract

**Background:**

There is a substantial variation in how different general practices manage knowledge implementation, including the degree to which activities are collectively and formally organised. Yet, it is unclear how these differences in implementation activities affect quality of care.

**Aim:**

To investigate if there are associations between specific formalised knowledge implementation activities and quality of care in general practices, exemplified by the use of spirometry testing.

**Design & setting:**

A nationwide cross-sectional study combining survey and register data in Denmark.

**Method:**

An electronic questionnaire was distributed to GPs, and data on spirometry testing among first-time users of medication against obstructive lung diseases were obtained from national registers. Associations were investigated using multilevel mixed-effect logit models.

**Results:**

GPs from 1114 practices (58%) responded, and 33 788 patients were linked to a responding practice. In partnership practices, the frequency of interdisciplinary and GP meetings affected the quality of care. Interdisciplinary and GP meetings held on a weekly basis were significantly associated with a higher level of quality of care and this was measured by the odds ratio (OR) of patients having spirometry. The development of practice protocols and standard recordings in the electronic medical record (EMR) for a range of disease areas compared with few or no areas at all also impacted the quality of care level provided. The effect of formalised implementation activities was not as evident in single-handed practices as in partnerships.

**Conclusion:**

This study provides valuable knowledge for GPs who aim to organise their practice in a way that supports implementation and quality improvement most effectively. Also, results may be useful for managers of implementation strategies and quality improvement initiatives when planning future activities.

## How this fits in

Implementing evidence-based knowledge in the daily care for patients is a fundamental prerequisite for delivering high-quality care in general practice. However, the time and resources spent on knowledge implementation activities vary substantially between practices. This study shows that, in partnership practices, formalised implementation activities in the form of frequent meetings and development of standardised processes of care are associated with quality of care. Furthermore, it demonstrates that such activities seem less important in single-handed practices.

## Introduction

Implementing evidence-based knowledge in the daily care for patients is a fundamental prerequisite for delivering high-quality care in general practice. However, despite many years of research into how to reduce the gap between evidence and practice, implementation still represents a significant challenge.^1^ Previously investigated implementation strategies, which have focused primarily on the individual practitioner, have overall shown only small to moderate effects.^2^ In recent years, organisational factors have become widely acknowledged as vitally important for ensuring successful implementation even though more knowledge is needed about which specific organisational factors affect implementation.^3^ This is important because a key element in securing high-quality care is to understand the effect of different factors on implementation.^4^

Novel research shows a substantial variation in how implementation of guidelines is managed in general practices, including the degree to which activities are collectively and formally organised.^5^ Implementation of evidence can be accomplished through formal or informal activities,^6^ and previous research indicates beneficial effects of specific organisational factors related to the degree of formalisation. In this respect, there has been a well-established consensus in the literature that professional interactions constitute a crucial part of implementation,^7–9^ and practice meetings are considered to be important.^10^ Furthermore, in qualitative and ethnographic studies, the effect of developing standardised processes of care, for instance practice protocols, has been associated with successful implementation and a high quality of care.^8,11–13^ However, within general practice, the evidence on the effects of meetings on implementation has been ambiguous,^14,15^ and no evidence exists from large-scale quantitative studies.

Even though general practice research indicates a positive effect on quality of care of formalised knowledge implementation activities, findings from the business literature suggest that a high degree of formalisation can exert a negative influence on concepts related to implementation, knowledge management, and knowledge performance.^16,17^ Since GPs have to prioritise their time and resources effectively, it is essential to investigate whether there is an association between formalised knowledge implementation activities and quality of care in general practice.

In order to do this, data that display variation in the delivery of evidence-based health care is required.^18^ The use of spirometry in the diagnosis of airflow limitation provides a useful example of an evidence-based recommendation^19,20^ that can be used as a proxy for quality of care, highlighting areas where substantial variation between practices was demonstrated.^21 ^Many patients who redeem first-time prescriptions for medication to treat obstructive lung diseases do not undergo spirometry testing^22^ and, in recent years, a general underutilisation of spirometry in the diagnosis of both asthma and COPD has been a consistent finding across countries.^23–28^ Variation has, to some degree, been explained by patient factors^29^ and organisational characteristics of the practices.^21^ However, considerable variation remains unexplained.

The aim of this study was to investigate if there are associations between specific formalised knowledge implementation activities and quality of care in general practices, exemplified by the use of spirometry testing.

## Method

### Setting

Denmark is a country with 5.7 million inhabitants of whom 98% are listed with a specific general practice. GPs work as private entrepreneurs on contract with the public funder and act as gatekeepers with regard to referrals to specialists and hospitals. All services are free of charge to patients, including spirometry.^30^ The majority of spirometry tests conducted among new medication users are performed in general practice by the GP or the practice staff.^22^ All general practices have access to spirometry, mainly in their own practice, but also by referral to hospitals or outpatient clinics. The practice units all have EMR.

All Danish citizens are assigned a unique personal identification number, which is registered in the Danish Civil Registration system.^31^ Likewise, each general practice is assigned a unique identification number. These identification numbers are used in national registers, enabling accurate linkage between patients, healthcare services, and general practices.^32^

### Design

A cross-sectional study combining questionnaire and register data was carried out. It covered all general practices in Denmark that had one or more GPs with an email address registered at the Danish Organisation of GPs. Also, data on spirometry testing among first-time users of medication against obstructive lung disease was obtained from national registers.

### Questionnaire

The development of the questionnaire was primarily inspired by the work of Gabbay and le May on how organisational features of the practice mediate clinicians’ refinement of mindlines.^8,9^ It was further qualified by qualitative interviews with seven GPs, who were purposefully sampled in order to cover a broad range of approaches towards implementation.^5^ Together with discussions in the research group, evidence on organisational innovation^33,34^ was used to inform the final selection of items to include. Besides covering specific practice characteristics, such as the level of task delegation to practice staff and status as training practice, the questionnaire comprised questions on three domains covering organisational factors related to formalisation of implementation activities. These were meeting structure, standardised processes of care, and task differentiation among GPs. Task differentiation implied that GPs in a practice had formally delegated the responsibilities for medical update in specific areas of disease between them. Testing of the questionnaire has been described elsewhere.^35^ Questionnaires were electronically distributed on 4 December 2013. One reminder was sent out on 7 January 2014 and the survey was closed on 20 February 2014. Participation was voluntary, and no financial compensation was given to responders.

### Register data

Patients for this study were selected from the Danish National Prescription Register.^36^ The selection criteria included patients who were aged ≥17 years in 2012 and who had redeemed a prescription for medication to treat obstructive lung disease defined by the anatomical therapeutical code (ATC) R03. In order to include only first-time users in the study, patients who had redeemed a prescription of R03 medication within the previous 5 years were excluded. For each patient, data on socioeconomic and demographic status was retrieved.

Information on the dates of spirometries was extracted from two separate registries: the National Health Service Register,^37^ which covers primary care, and the National Patient Register,^38^ which covers hospitals and outpatient clinics.

Along with the email addresses, the Organisation of Danish GPs provided information on the unique identification number of each general practice, the practice form, as well as GPs’ age and sex.

### Explanatory variables

GPs’ answers to the questionnaire were pooled on practice level and used as explanatory variables. If disagreement between GPs in the same practice occurred, the highest level of formalisation reported was used. Box 1 displays how domains in the questionnaire were operationalised into explanatory variables in the analysis.

**Box 1.** How domains in the questionnaire were operationalised into explaining variables in the analyses

Domains	Items	Description	Dichotomisation
**Meeting structure**	Scheduled meetings	The occurrence of scheduled meetings (versus ad hoc conversations)	Scheduled meetings: Yes/no
	Interdisciplinary meetings	Frequency of GP and staff meetings: *Weekly/monthly/quarterly/rarer/never*	Weekly: Yes/no
	GP meetings	Frequency of sole GP meetings: *Weekly/monthly/quarterly/rarer/never*	Weekly: Yes/no
	Educational meetings	Frequency of meetings aimed at learning about a specific topic: *Weekly/monthly/quarterly/rarer/never*	Weekly: Yes/no
	Formalised meetings	A formal agreement about agenda, mediator and/or minutes in relation to meetings. For each factor is stated: *Always/often/sometimes/rarely/never*	Always for all factors: Yes/no
**Standardised processes of care**	Practice protocols	The extent to which practice protocols are developed in the practice: *In a range of areas of disease/in a few areas of disease/none at all*	In a range of areas of disease: Yes/no
	Standard laboratory requisition formulas	The extent to which standard laboratory requisitions formulas are developed in the practice: *In a range of areas of disease/in a few areas of disease/none at all*	In a range of areas of disease: Yes/no
	Standard phrases in the EMR	The extent to which standard phrases in the EMR are developed in the practice: *In a range of areas of disease/in a few areas of disease/none at all*	In a range of areas of disease: Yes/no
**Task differentiation among GPs**	Responsibilities for medical update	The extent to which responsibilities regarding medical update (on for instance COPD, DM and IHD) are based on a formal agreement in the practice: *yes/informal/no*	Formal agreement: Yes/no

COPD = chronic obstructive pulmonary disease. DM = diabetes mellitus. EMR = electronic medical record. IHD = ischemic heart disease.

### Outcome variable

For each first-time user of R03 medication, it was observed whether a spirometry was performed in an observation period that ran from 6 months prior to the first prescription redemption until 12 months after.^21^

### Statistical analyses

Multilevel mixed-effects logit models were used with patients nested within practices to calculate ORs with 95% confidence intervals (CIs) for the associations between formalised implementation activities and patients having spirometry performed.

Previous research has demonstrated associations between specific practice and patient characteristics, and spirometry testing,^21,22,29^ and these factors were adjusted for in the analyses. Practice characteristics comprised: GPs’ age and sex, status as training practice (yes/no), and task delegation to practice staff. Task delegation was dichotomised into delegation of follow-up care of chronic diseases: yes/no. Patient characteristics comprised: age, sex, income, highest attained education, labour market affiliation, cohabitation status, and severe disease: yes/no. Severe disease was defined as repeat redemptions of R03 medication and initiation of more than one type of R03 medication within the first year. ORs are presented with 95% CI in the tables. *P*<0.05 was considered statistically significant.

Based on a hypothesis that the effect of formalised implementation activities on patients’ OR of having spirometry performed would differ among practice forms, analyses were stratified into single-handed and partnership practices. Initially, in the analyses, partnership practices were further divided into small (2–3 partners) and large partnerships (>3 partners). However, no noteworthy differences between small and large partnerships could be inferred. The two groups were therefore collapsed in order to obtain higher power in the analyses. STATA (version 14.1) was used for all statistical analyses.

## Results

**Figure 1.**
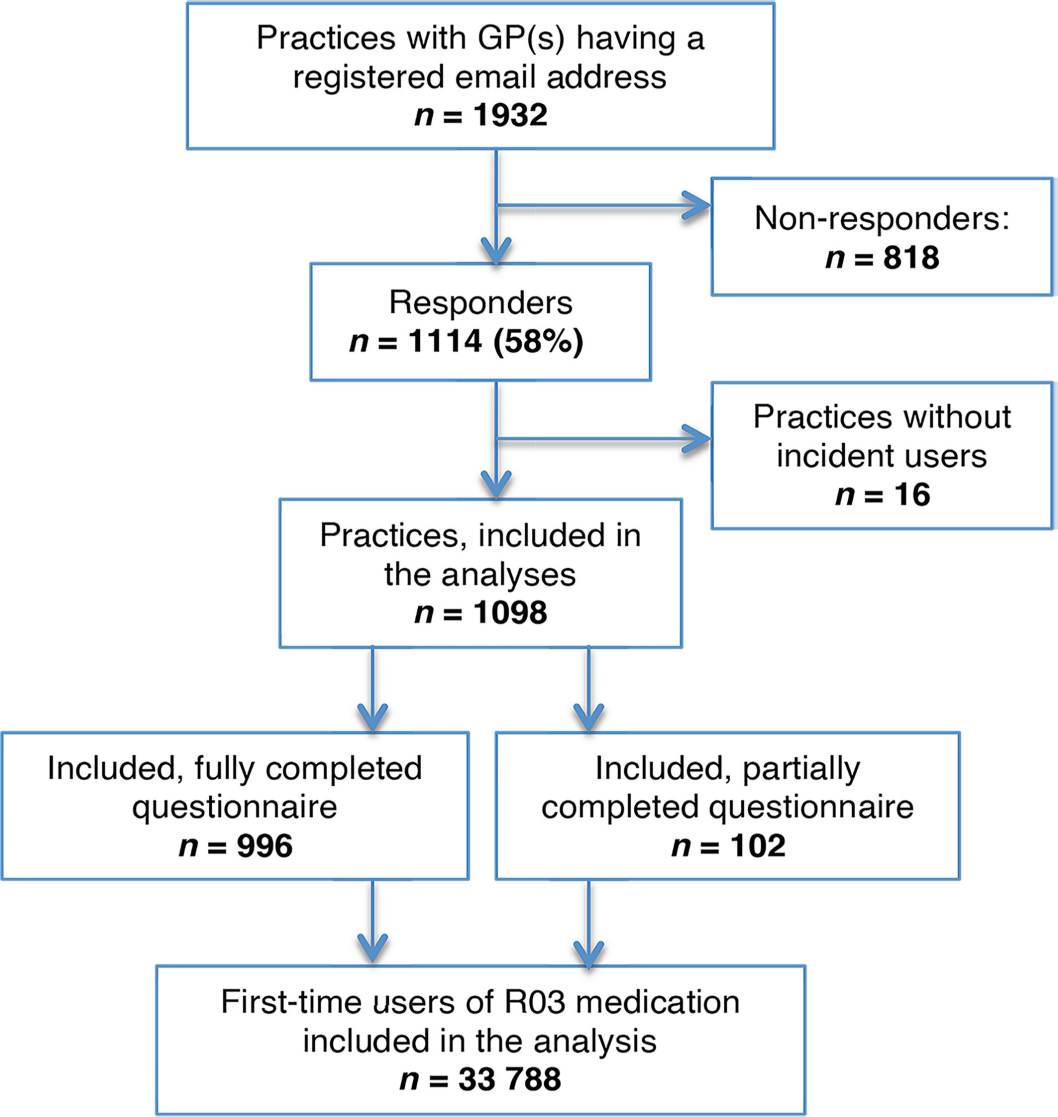
Flowchart of the study population.

Out of the 2117 general practices identified in the National Health Service Register, 1932 (91%) had one or more GPs with an email address registered at the Danish Organisation of GPs. GPs from 1114 practices (58%) responded, of which 476 (43%) answers came from single-handed practices and 638 (57%) from partnerships. GPs from 996 practices (52%) answered all questions in the questionnaire. A total of 56 269 first-time users of R03 medication were identified. After excluding the patients where the regular GP could not be identified or did not respond to the questionnaire, those who died or migrated during the study period, and those with missing sociodemographic data, 33 788 patients were linked to a responding practice (Figure 1).

With the exception of educational meetings, a significantly higher proportion of formalised implementation activities in partnership practices was found compared to single-handed practices (Table 1).

**Table 1.** Distribution of implementation activities in single-handed and partnership practices

	Single-handed, *n* (%)	Partnership, *n* (%)	*P*-value^a^
**Meeting structure**			
Scheduled meetings — Yes	249 (60.4)	544 (92.4)	<0.001
Interdisciplinary meetings — Weekly	41 (16.6)	161 (29.6)	<0.001
GP meetings^b^ — Weekly	30 (25.2)	243 (44.7)	<0.001
Educational meetings — Weekly	37 (15.0)	101 (18.6)	0.220
Formalised meetings — Yes	55 (22.3)	211 (38.8)	<0.001
**Standardised processes of care**			
Practice protocols — In a range of disease areas	153 (37.5)	394 (67.0)	<0.001
Standard laboratory requisition formulas — In a range of disease areas	219 (53.7)	473 (80.4)	<0.001
Standard recordings in the EMR — In a range of disease areas	131 (32.1)	303 (51.5)	<0.001
**Task differentiation among GPs**			
Formal agreement^b^ — Yes	30 (19.4)		<0.001

^a^Calculated using χ^2^. ^b^GPs in single-handed practices were only asked these questions if they had reported being in collaboration with other practices. EMR = electronic medical record.

**Figure 2.**
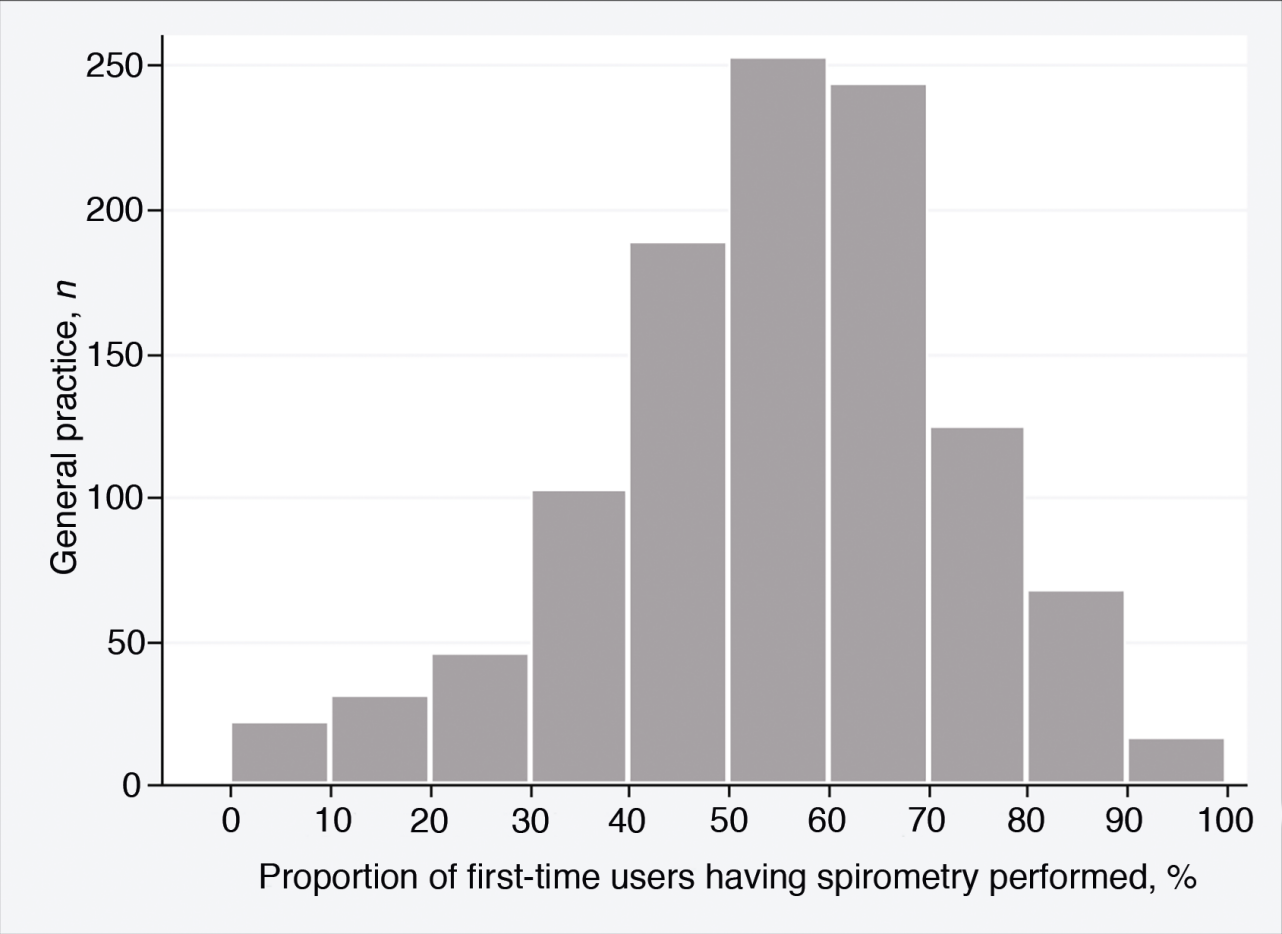
Variation in spirometry proportion among general practices.

Among general practices, the mean proportion of first-time users of R03 medication who had a spirometry performed was 55%. Figure 2 demonstrates the variation between practices.

After adjusting for practice characteristics (GPs’ age and sex, status as training practice and task delegation to practice staff) and patient characteristics (age, sex, income, education, labour market affiliation, cohabitation status, and disease severity) the most pronounced effects of formalised knowledge implementation activities on patients’ ORs of having spirometry performed were found in partnership practices. In single-handed practices, it was only the development of standard laboratory requisition formulas in a range of disease areas that showed significant association with patients’ OR of having spirometry performed. In partnership practices, weekly interdisciplinary meetings and GP meetings (as opposed to rarer meetings) as well as development of practice protocols and development of standard recordings in the EMR in a range of disease areas (as opposed to a few areas or none at all) were significantly associated with patients’ ORs of having spirometry performed (Table 2). It should be noted that, except from OR on formalised meetings and task differentiation (only single-handed practices), all ORs pointed towards a positive effect on spirometry.

**Table 2.** Associations between implementation activities and patients having spirometry performed

	Single-handed		Partnership	
	*n* (%)	OR adjusted (95% CI)	*n* (%)	OR adjusted (95% CI)
**Meeting structure**				
**Scheduled meetings** No Yes	1296 (49.6) 2343 (54.5)	1 1.09 (0.89 to 1.33)	796 (54.1) 12 671 (55.3)	1 1.00 (0.82 to 1.22)
**Interdisciplinary meetings** Monthly or rarer Weekly	1903 (53.2) 423 (60.3)	1 1.20 (0.89 to 1.61)	8863 (54.8) 3808 (56.7)	1 1.12 (1.00 to 1.25)^b^
**GP meetings^a^** Monthly or rarer Weekly	801 (51.0) 285 (56.2)	1 1.40 (1.00 to 1.96)	6773 (53.6) 5898 (57.4)	1 1.15 (1.04 to 1.27)^b^
**Educational meetings** Monthly or rarer Weekly	1924 (53.9) 402 (57.0)	1 1.06 (0.79 to 1.43)	9928 (54.8) 2743 (57.6)	1 1.05 (0.92 to 1.20)
**Formalised meetings** No Yes	1817 (54.8) 509 (52.9)	1 0.85 (0.65 to 1.10)	6998 (55.1) 5673 (55.7)	1 1.00 (0.90 to 1.11)
**Standardised processes of care**				
**Practice protocols** Few/none at all In a range of disease areas	2100 (50.5) 1481 (55.3)	1 1.13 (0.93 to 1.37)	3670 (50.3) 9783 (57.4)	1 1.29 (1.15 to 1.43)^c^
**Standard laboratory requisition formulas** Few/none at all In a range of disease areas	1459 (48.5) 2122 (55.4)	1 1.28 (1.06 to 1.55)^b^	2054 (52.7) 11 399 (55.8)	1 1.13 (0.99 to 1.29)
**Standard recordings in the EMR** Few/none at all In a range of disease areas	2324 (51.3) 1257 (54.6)	1 1.02 (0.84 to 1.25)	6017 (53.6) 7436 (56.7)	1 1.14 (1.03 to 1.26)^b^
**Task differentiation among GPs**				
**Formal agreement^a^** Informal/no Yes	1073 (50.7) 296 (49.7)	1 0.99 (0.71 to 1.39)	7795 (54.1) 5658 (56.9)	1 1.10 (1.00 to 1.23)

ORs are adjusted for practice characteristics (GPs’ age and sex, status as training practice, task delegation to practice staff) and patient characteristics (age, sex, income, highest attained education, labour market affiliation, cohabitation status, and severity of disease).

^a^GPs in single-handed practices were only asked these questions if they had reported being in collaboration with other practices. ^b^ *P*<0.05. ^c^ *P*<0.001. EMR = electronic medical record. OR = odds ratio.

## Discussion

### Summary

In partnership practices, weekly GP and interdisciplinary meetings, and development of standardised processes of care in a range of disease areas were associated with higher quality of care measured by patients’ ORs of having spirometry performed in relation to initiation of R03 medication. Development of practice protocols showed the strongest association (*P*<0.001). The effect of formalised implementation activities was not as evident in single-handed practices.

### Strengths and limitations

The use of data from national registers is a major strength of this study, and the validity of the national registers is generally considered high.^32^ All R03 medications require a prescription, and registration of first-time users is therefore virtually complete. Likewise, a prerequisite for providers being reimbursed when performing a spirometry is that it is recorded in the registers. This offers a strong incentive for providers to report spirometric procedures. The register data enabled adjustment for a range of relevant patient characteristics^29^ that, together with adjustments for practice characteristics, allowed for further isolation of the associations between formalised knowledge implementation activities and spirometry testing.

It may have been relevant to include other practice characteristics in the analyses, for instance primary care physician supply. This specific characteristic has previously been associated with quality of care for chronic conditions.^39^ However, the authors did not have access to reliable data on this measure and, specifically regarding spirometry testing among first-time users of R03 medication, previous research has not shown an association.^21^

This study's finding on mean spirometry proportion among practices is higher than that previously reported in a similar setting (55 versus 50.8%, see Table 2).^21^ The difference may partly be explained by selection bias. In this study, spirometry proportion was calculated for GPs who chose to participate in the survey, whereas the comparator study included all GPs in the country. However, after making an additional calculation of these register data including patients of both responding and non-responding practices, the spirometry proportion did not change much (53%), which indicates a general increase in the use of spirometry.

The development of the questionnaire was based on previously published research and strengthened by qualitative interviews with the target group. This type of mixed-method approach has been described as the ‘instrument design model’.^40^ It provided the opportunity to qualify the choice of factors to include and thereby grounding it in the real-life clinical practice of participants.

Compared to the distribution of single-handed and partnership practices in the invited practice population (58 and 42% respectively), 43% of the responses were obtained from single-handed practices and 57% from partnerships. Single-handed practices were thus underrepresented among the responding practices. However, for partnership practices to be included, only one GP had to have responded, which can explain the somewhat skewed distribution of responses among practice forms.

A well-known limitation in survey research is that self-reported data involves the risk of introducing reporting bias. Despite the authors' efforts to provide as concrete questions as possible, there is some evidence of reporting bias as not all GPs in partnership practices gave the same answers to all of the questions. Since the highest level of formalisation stated in the analysis was used, the results reported in the present study may be a conservative estimate on the association between formalised implementation activities and spirometry testing.

### Comparison with existing literature

This study's results contribute to the literature by showing a positive association between weekly meetings and quality of care. Having a forum for sharing and discussing information and developing one’s knowledge is essential for successful implementation,^7,8,11^ and it has been speculated that informal interactions might be more important than formal ones in establishing interactions that are easy and constructive.^9^ Regarding implementation, this study's results underline the importance of also having a formalised forum, particularly in partnership practices, where knowledge can be shared and discussed if implementation is to be successful.^7,8^ Furthermore, it appears important that interactions occur frequently as the mere occurrence of scheduled meetings did not reveal any association with quality of care. These findings support qualitative research findings on characteristics of practices that succeed in quality improvement.^41^ In concurrence with findings from the business literature on the negative effect of a high degree of formalisation,^16,17^ having a formalised meeting structure (agenda, mediator and minutes) showed no effect. It seems possible that the translation process of implementation will occur at meetings even if no formalised structure is present and, taking findings from the business literature into account, it might be expected that, in some cases, it could lead to a freer discussion.

Developing practice protocols or other standardised processes of care is a way of ensuring consistency in the approach to patients within a practice. However, the most important features of such processes are presumably the reflections and discussions required to ensure their applicability to the local context; for example, consideration of how new recommendations fit into existing knowledge, skills and expertise, practice routines as well as the perceived impact on remuneration and quality of care.^9^ Such discussions can then lead to a practical interpretation of who does what and when and how, which is necessary for developing a practice protocol. Using a quantitative design, the current study confirms findings from previous qualitative studies showing a positive effect of such processes on quality of care.^8,11–13^ It also corresponds to previous quantitative research that indicated the same effect; although those conclusions were based on self-reported outcome measures and included fewer responders.^42^

Having an anchor person who takes on responsibility for improvement^15^ or a formally appointed internal implementation leader^6^ has been proposed to influence implementation and quality improvement positively. Similarly, task differentiation among GPs has been associated with spirometry utilisation.^42^ This study's results were unable to confirm these findings. The lack of effect was surprising, but could be explained if a lack of explicit time was dedicated to the task,^6^ or by a lack of commitment of the person in question.^15^ However, since the survey was not aimed specifically at obstructive lung diseases, it could also be that not all practices that reported having formalised task differentiation had a person responsible for this specific area.

Even though the results pointed in a clear direction of a positive effect of specific formalised implementation activities in both single-handed and partnership practices, the effect sizes may not be that pronounced. For significant results, it ranged from 12% (interdisciplinary meetings) to 29% (practice protocols). This underlines that there are many other factors that influence implementation ranging from characteristics of the implementation object, such as guidelines to characteristics of the individual practitioner, and to the organisation and beyond.^3^

By their very nature, single-handed practices are different from partnership practices. This probably explains the differences on the effect of meetings since single-handed practices might not require these as often. Previous research has not been able to demonstrate a higher quality of care in partnership practices than in single-handed practices, neither generally^43^ nor specifically concerning spirometry testing.^21^ This, together with the current results, suggests that in order to maintain a high quality of care in more complex organisations, such as partnership practices, formalisation of implementation activities is required to a greater extent.

### Implications for research and practice

GPs have to prioritise their time and resources effectively, and having frequent meetings and developing standardised processes of care are both time and resource consuming. However, these results suggest that such formalised implementation activities matter to quality of care, especially in partnership practices, and could therefore be worthwhile. Agreeing on collective norms and targets for improving care and creating space and a respectful dialogue have been suggested to be some of the essential elements in fostering successful transformation of clinical knowledge.^44^ These attributes are presumably closely related to the effect of formalised implementation activities on quality of care that were found in this study and should be kept in mind for quality improvement purposes. These results, though, do not allow for conclusions about causal relationships to be drawn and future research should investigate the effects in an experimental design. Furthermore, it would be relevant to investigate if the results can be applied to other evidence-based recommendations than spirometry testing. 

Frequent meetings and the use of standardised processes of care are associated with higher quality of care, measured by spirometry testing, in partnership practices. The effect of these formalised implementation activities in single-handed practices is less evident. 

This is valuable knowledge for GPs who aim to organise their practice in a way that supports implementation and quality improvement most effectively. Also, results may be useful for managers of implementation strategies and quality improvement initiatives when planning future activities.
